# Removal of denture adhesives from PMMA and Polyamide denture base materials

**DOI:** 10.1590/1678-7757-2020-0448

**Published:** 2021-03-26

**Authors:** Nick POLYCHRONAKIS, Nikitas SYKARAS, Gregory POLYZOIS, Panagiotis LAGOUVARDOS

**Affiliations:** 1 National & Kapodistrian University of Athens School of Dentistry Department of Prosthodontics Athens Greece National & Kapodistrian University of Athens, School of Dentistry, Department of Prosthodontics, Athens, Greece.; 2 National & Kapodistrian University of Athens School of Dentistry Department of Operative Dentistry Athens Greece National & Kapodistrian University of Athens, School of Dentistry, Department of Operative Dentistry, Athens, Greece.

**Keywords:** Denture adhesives, Denture cleansers, Removal methods, PMMA, Polyamide

## Abstract

Denture adhesives need complete removal due to their frequent replacement.

**Objective:**

Our study investigates the removal of denture adhesives from denture base materials, using different methods.

**Methodology:**

PMMA and Polyamide denture base materials were used to fabricate 120 samples (15×15×1.5mm). One side of the samples was left as processed and the other polished with a usual procedure, hydrated for 24 h, dried, and weighted. They received 0.2 g of three adhesive creams on their unpolished surface (Corega, Olivafix, Fittydent), pressed on polysulfide material, stored under 37°C and 95% rel. humidity for 1 h and 60 of them, following their separation from polysulfide base, brushed under running water, whereas the rest inserted in a cleanser bath (Fittydent Super) for 5 min. The samples were dried and inserted in the oven (37°C) for additional 10 min and weighted again. Roughness tests of denture materials and light microscopy of adhesives creams were also used to evaluate the materials. Time lapse images of spayed with water adhesives on PMMA base were also taken to evaluate the volumetric changes of adhesives. Weight data before and after adhesive removal, indicating the amount of remaining adhesive, were statistically analyzed using Welch’s ANOVA and Games-Howell multiple comparisons tests at α=0.05 level of significance.

**Results:**

Roughness of Polyamide was higher than PMMA and Fittydent showed greater volumetric changes than the others. Significant differences (p<0.05), were found between PMMA and Polyamide bases, between Olivafix and Fittydent adhesives, and between brushing and cleansing methods but only for PMMA-Olivafix combination.

**Conclusions:**

Adhesives showed a stronger adherence to PMMA surface, and Fittydent was the most difficult to be removed. Removal methods were not effective for all adhesives or denture base materials. These indicate that removal methods, adhesive type and denture base material are all playing a significant role in the removal of adhesives from denture surfaces.

## Introduction

Complete dentures have historically been the standard treatment for complete edentulous patients with or without the use of dental implants. In most cases the materials used for denture base fabrication are the heat-cured polymethyl-methacrylate (PMMA) due to its physico-mechanical properties. However, in some cases i.e. allergic patients to residual monomer, other materials, including polyamides are proposed.^1^

In general, maxillary edentulous patients are satisfied with the use of complete dentures. Edentulous patients in the mandible, on the other hand, express various complaints with their denture and a general dissatisfaction mainly due to the lack of retention.^2,3^One of the methods to overcome this problem is the use of natural or synthetic denture adhesives in the form of powder or cream, gel and strips.

Denture adhesives act as intermediate substances between the intaglio surface of a denture and the underlying mucosa. In fact, these materials are made of synthetic polymers with a short or long action that upon hydration (presence of saliva) increase in volume and fill the space between the two surfaces, enhancing retention due to exclusion of air and saliva.^4^Moreover, free carboxyl groups form ionic adherence assist in the increase of interfacial forces and therefore the retention of dentures is enhanced.

All forms of adhesives contain a main adhesive component (5%-60% bw), a water-insoluble component (20%-70% bw), viscosity index improvers (1%-20% bw), plasticizing agent (1%-10% bw), gallant agent (1%-10% bw), and possibly therapeutic and sensate additives for flavor and fragrance.^5^The main adhesive component (mainly salts of alkyl vinyl ether-maleic anhydride-AVE-MA) is muco-adhesive, hydrophilic and water-soluble that expands upon hydration.^5^The water-insoluble component (mainly waxes, petrolatum, oils, silicone, PolyVinylAcetate) contributes to the cohesion of the product since it swells less than 10% in water. The viscosity index improver (PolyMethylAcrylate, acrylic resins, PolyVinylChloride, nylon, polyesters) regulates the overall viscosity of the product to behave normally within temperature changes in the mouth. Plasticizing agents are water-insoluble (polyols, glycerin, propylene glycol, xylitol) and are used for softening the product. To further increase the action of the products (long-acting polymers), cohesive forces are enhanced via molecular cross-linking, increasing the overall adhesive properties of the materials and the resistance of denture removal.^6^

Denture adhesives have a positive effect on denture retention and mastication^7-11^and improve the quality of life of users.^12^However, their retentive forces are varying among the materials,^13,14^and negatively affect the retention of milled complete dentures.^6^

Although most people are satisfied with the use of denture adhesives, their daily (or twice daily) use create a problem for the incomplete removal from dentures and oral tissues.^13,15-18^The complete removal from dentures is important for the hygiene^19^ and the stability of the denture, since every new layer of adhesive over remnants of an old one increases the vertical dimension of occlusion^20^ and increases the biofilm and the formation of reservoirs of potentially infectious pathogens that may affect the health of oral tissues.^21,22^However, the information on this issue is controversial; Ozkan, et al.^23^(2012) and Leite, et al.^24^ (2014), for example, conducted a 2 months and a 15 days clinical studies, respectively, and reported that the denture adhesives do not impair the oral microbiota.

Thus, a global task force was established within Oral Health Foundation to discuss and develop guidelines for the use of denture adhesives which resulted in a very important white paper.^25^

To remove adhesives from the denture intaglio surface, manufacturers propose brushing dentures after immersion in a warm water bath or under running water, whereas others suggest the combined action of a brush with a denture cleanser.^26-29^ Many studies^26,27,29^have concluded that the proposed methods of denture adhesive removal drastically decrease the amount of the residual adhesive from the denture intaglio surface, but not completely. Three methods of adhesive removal can be identified in the literature: the mechanical (use of a brush), the chemical (use of water, soap solution or denture cleanser), and their combination. The brushing method removes most of the material from the surface, but combined with a cleanser it seems to reduce even more the adhesive residues.^27^ However, none of these studies directly compared brushing action to the action of a cleanser alone, since it was always combined with the brushing. The use of a cleanser was mainly introduced to eliminate the bacterial load from the denture and it was proven to be more effective than the brushing alone.^30^Nevertheless, the question of whether a cleanser alone is capable of removing adhesive remnants as effectively as a brush still remains and is very important for the adult people, who are the main denture wearers.

Another point that attracts attention is that all previous studies^26-29^along with some others^31,32^evaluated the remaining adhesive on the surface of denture base materials by visual methods, as recommended by the washability test.^33^Creams are usually transparent and colorless, or pink materials, which make difficult the identification of small amounts on a pink denture surface even if they are colored differently from the base or using sophisticated image processing methods.^29^ This makes visual methods not the most efficient ones for the recording of small amounts of adhesives,^34^ even though they are recommended in ISO.

Finally, since no studies have reported on the use of adhesives on polyamide denture base materials, there is a question if the adhesive is as efficient on such surfaces as on PMMAs.

Therefore, the aim of our study was to evaluate the efficacy of two basic methods (use of a brush or a cleanser) for the removal of several adhesives that are applied on a PMMA or Polyamide denture base material. The null-hypothesis was that the methods, denture base materials, and adhesives present no significant differences in the removal of the adhesive layer.

## Methodology

**Figure 1:**
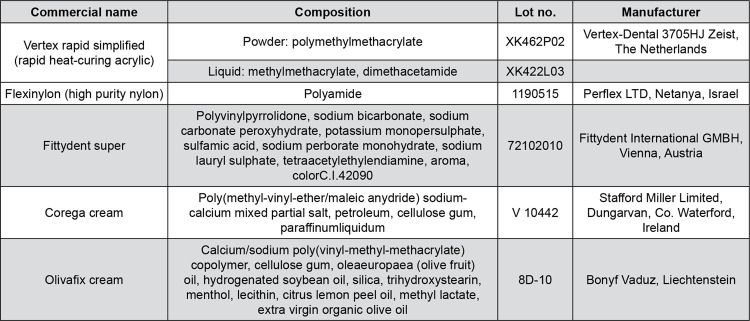
Commercial name, composition and manufacturer of the materials used in the study

**Figure 2:**
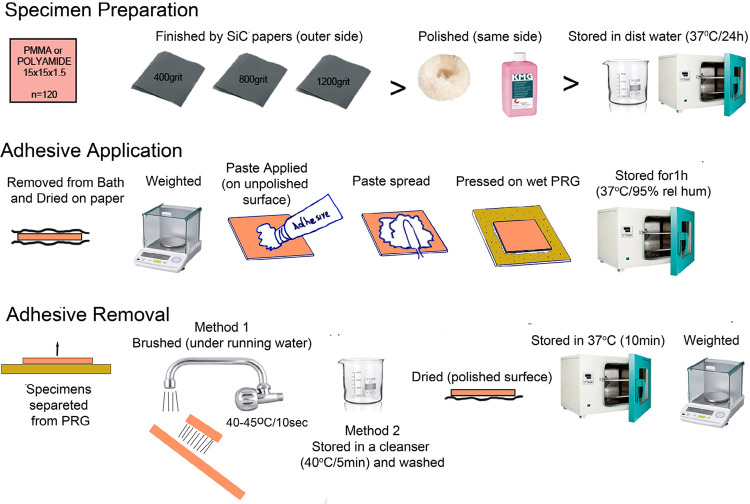
Schematic representation of the weighting methodology process, divided in three distinct phases. Specimen preparation, adhesive application and adhesive removal (PRB is for polysulfide rubber base).

For the study, 60 rectangular specimens of 15×15×1.5 mm were made of PMMA and 60 from Polyamide material (120 in total). Manufacturer, material type and compositions are shown in Figure 1 and the methodology followed is graphically represented in Figure 2. In the case of PMMA specimens, the conventional denture flasking technique was followed, whereas in the case of Polyamide ones an injection-molding technique was applied. Both techniques were used according to the manufacturers’ recommendations. Specimens were finished on one of their surfaces using successive grits of wet or dry SiC papers (400, 800, 1200 grit) and then polished using polishing liquid (KMG; Candulor AG, Zurich, Switzerland) on a white cotton yarn wheel polishing brush (Bur Dental; Guangzhoo Co. Ltd, Guangzhoo, China). The other surface of the specimen left as processed by its usual heat and pressure or injection denture construction process protocol.

Specimens were stored in distilled water within a dry oven of 37**°**C for 24 h before the measurement of their weight and the application of an adhesive. A 24 h hydration in distilled water was considered enough for the specimen, used also in other experiments, despite the different hydration time for all base materials. After their removal from the bath, specimens’ surfaces were dried, dabbing them on a soft paper tissue and their weight was measured within the next one minute. This was based on time lapse tests of several samples which showed a loss of 0.0001-0.0003 g but after 3 min.

The samples were weighed by the same, experienced in the technique person, on a digital analytical balance measuring four decimal places (AW220; Shimadzu Corporation, Kyoto, Japan). Before measurement, their surfaces were dried, dabbing them on a soft paper tissue.

### Adhesive Application

After measuring the weight of the specimens, 0.20 g of a denture adhesive cream (Corega/ Stafford Miller Ltd., Olivafix/Bonnyf Vaduz, and Fittydent/Fittydent Int.) was applied on the center of specimen’s unpolished surface and pressed on a thick polysulfide rubber impression material (Permlastic Regular body/ Kerr, Orange, California, USA) of 20**×**20 mm dimensions and 2 mm thickness that was sprayed with water, to simulate the oral mucosa.^35^Specimen pairs (PMMA or Polyamide with the polysulfide base) were then inserted in an oven of 37**°**C and 95% relative humidity, for 1 h.

### Adhesive Removal

Specimen pairs were removed from the oven and pulled apart on a vertical direction. The denture base rectangle was separated from the polysulfide rubber base.

Method 1: A soft brush with ultra-thin ending bristles (Clinic Gum Protector, Jordan, Orkla Lilleborg AS, Oslo, Norway) was used under 45-degree running warm water, for 10 back and forth movements (lasting about 10 s) with a pressure close to 400 g. The ability of the brush to bend in forces larger than 400 g helped in keeping the forces below but close to this level. Water run, dropping distance, specimen’s inclination and number of strokes were always the same and performed by the same researcher. All brushings were made by the same person experienced in the technique to keep the same water run, dropping distance, specimen’s inclination and number of strokes the same for all specimens.

Method 2: A second group of 60 specimens were used to test the second method of adhesive removal. The methodology was the same as the previous one except that brushing action was replaced by a denture cleanser. After their removal from the polysulfide surface, the specimens were placed under running warm water for 10 s, and then they were immersed for 5 min in a water bath of 250 mL at 40-45**°**C, with a tablet of a cleanser in it according to manufacturer (Fittydent Super/Fittydent International GMBH, Vienna, Austria).

The specimens’ polished surface was dried on a soft tissue paper and then placed in an oven (37**°**C) for 10 min to dehydrate the remnants of the adhesive before the second measurement of its weight.

### Denture base roughness measurement

To understand the role of denture base material roughness on the resistance of adhesive removal, a non-contact optical interferometric profilometer (Wyko NT1100, Veeco) was used on 3 randomly and blindly chosen specimens from each material. The instrument was operated under the following conditions: vertical scan image mode Myro lens (5×2 FOV), 20.4× total magnification to include as much specimen area as possible in roughness calculations, 10 μm back scan length, 30 μm scanning length, and a modulation length of 2.

### Adhesive cream micro-imaging

Fresh adhesive creams, pressed between two clean cover glass plates, were photographed under a light microscope (DM4000B, Leica Microsystems, Wetzlar, Germany) operated in reflectance illumination mode at a 5× magnification.

### Imaging of hydrated adhesives

This small illustrative test was used to record possible differences in the water uptake and subsequent volumetric changes of the materials. The test used a PMMA bar of 3×15×60 mm dimensions, made and hydrated exactly as specimens for the main experiment. The amount of 0.2 g from all adhesive creams was applied on the bar and creams were immediately sprayed once with distilled water from a 6 cm distance, using a small water bottle sprayer. The bar was put in an oven of 37°C and 95% relative humidity for 180 min, and removed only at 10 min, 60 min and 180 min intervals for the imaging under an OlympusE-M10 Mark II digital camera (Olympus Corp, Tokyo, Japan).

### Statistical Analyses

Collected weighting data were statistically analyzed for differences in the efficacy of methods, and materials of removing the adhesives from denture base materials. Welch’s one way ANOVA with Games-Howell multiple comparisons tests were used at α=0.05 level of significance, using IBM-SPSS statistics v.25 (IBM Corp., Armonk, New York, USA).

## Results

**Figure 3:**
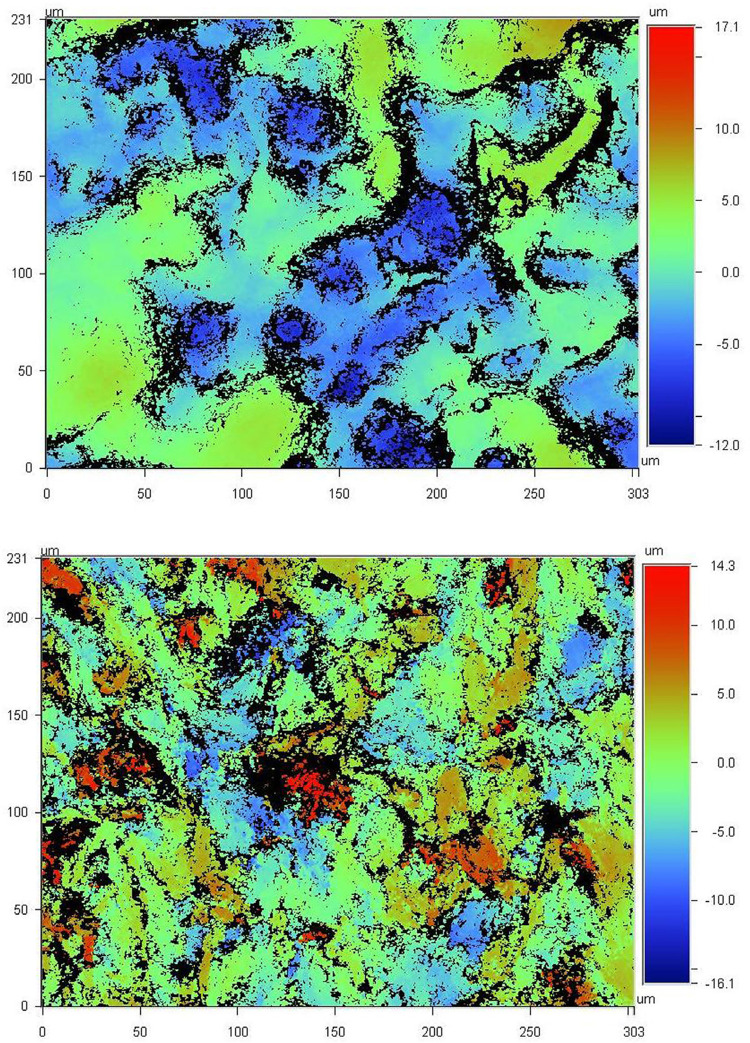
Vertical scan images by a non-contact optical interferometric profilometer at 20.4× magnification of PMMA (upper) and Polyamide (lower) denture base material with Ra values of 2.58 nm and 3.01 nm, respectively. Cool colors indicate depth and warm colors height of the areas in respect to 0 level of the surface

Profilometric data indicated lower mean Ra levels for PMMA (2.72±0.2 nm) than Polyamide materials (3.43±0.3 nm). Figure 3 shows representative vertical scan images of the measured areas at 20.4× magnification.

**Figure 4:**
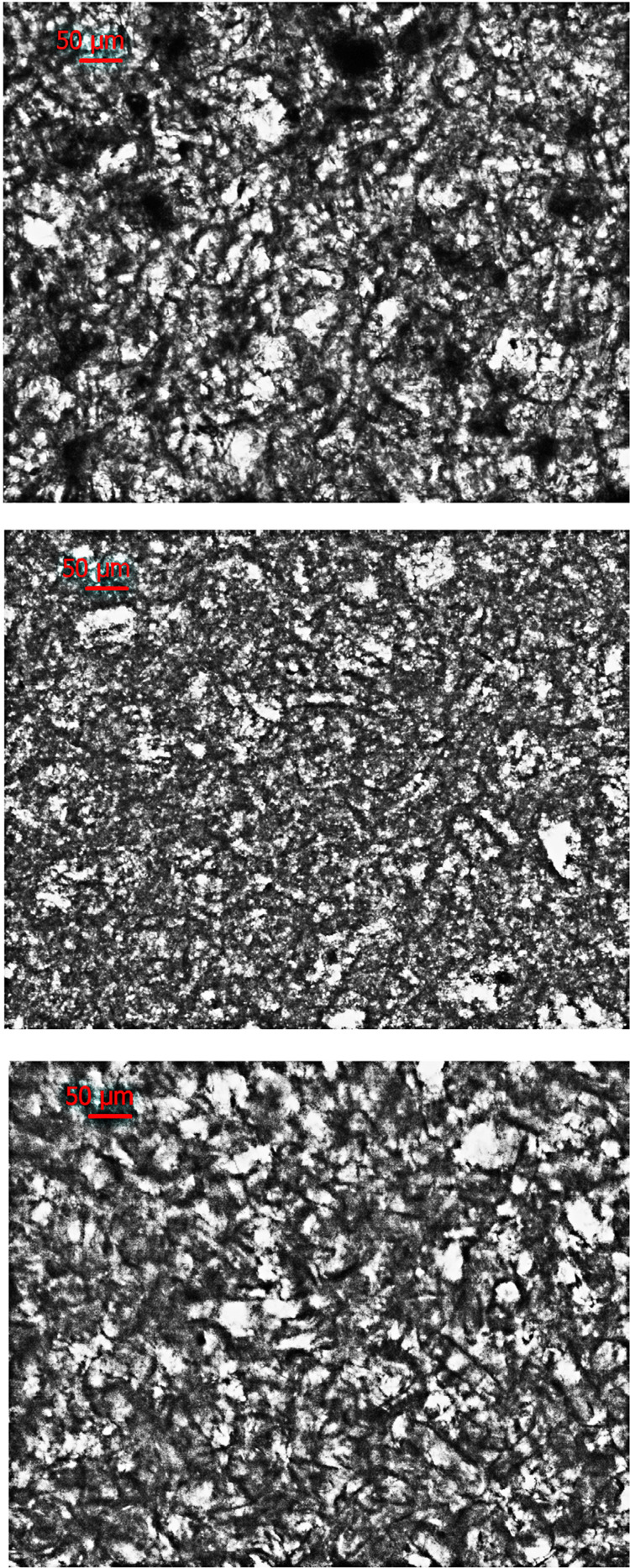
Microscopic images at 5× magnification of adhesive materials under reflectance illumination mode. In Corega cream (upper) we see the sodium-calcium poly(methyl-vinyl-ether/maleic anhydride) mixed salt in cellulose gum with lots of voids. In Olivafix cream (middle) we see the calcium/sodium poly(vinyl-methyl-methacrylate) copolymer in cellulose gum and in Fittydent cream (lower) agglomerations of silica particle in polyvilylacetate and sodium carboxymethylcellulose

Figure 4 shows optical microscopy images of the adhesive materials used in our study. At this magnification, their structure (mainly the polymeric hydrogel salts) look similar to each other.

**Figure 5:**
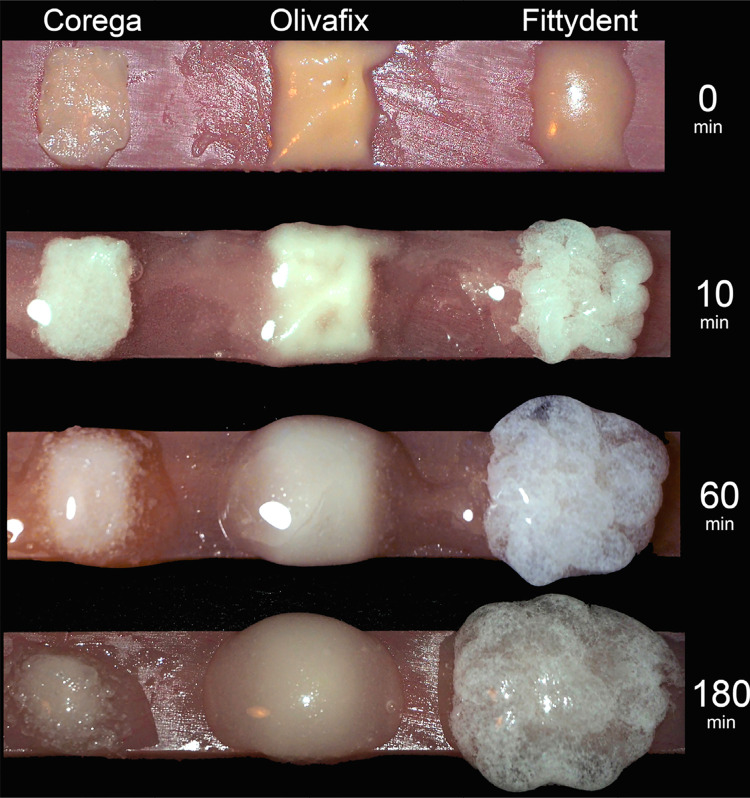
Photos of adhesives on a PMMA bar at 0 min, 10 min, 60 min and 180 min after their wetting with a water spray

Figure 5 shows time-lapse images of the hydrated adhesives over a PMMA surface. These indicate that all materials expand upon hydration, but Fittydent more than the others. At 60 min a significant part of Corega and Olivafix show signs of liquification (transparent areas) whereas Fittydent continues to expand even after 180 min with no signs of liquification.

Table 1 shows overall differences in the weight between initially hydrated samples of denture materials (Polyamide and PMMA) before the application and after the removal of adhesives by two different methods (brushing or cleansing). Fittydent shows higher values than the other two adhesives, on both denture base materials and for both methods. On PMMA the values of Fittydent are much higher than Polyamide. Corega gives values that are similar for methods or denture base materials but Olivafix seems to behave differently on the denture materials by the two methods.

**Table 1:** Means ± SD weights (g) of the adhesives left on specimen’s surface after their removal by the brush or the cleanser method

Removal	Denture	Denture Adhesives		
Method	Material	Corega	Olivafix	Fittydent
Brush	Polyamide	0.016±0.019_a/a_	0.001±0.001_a/a_	0.090±0.036_b/a_
	PMMA	0.010±0.006_a/a_	0.035±0.022_a/b_	0.184±0.039_b/b_
Cleanser	Polyamide	0.002±0.001_a/a_	0.001±0.001_a/a_	0.075±0.027_b/a_
	PMMA	0.013±0.009_a/a_	0.004±0.004_a/a_	0.236±0.031_b/b_

Note: Same letters before slash indicate not significant differences (p>0.05) among cells in the same raw and after slash among cells in the same column (n=10).

Normality test (Kolmogorov-Smirnov) indicated that all groups came from normally distributed populations (p>0.05), whereas Levene’s test showed unequal variances (p<0.0001). Welch’s one-way ANOVA test showed significant differences among groups (p<0.001) which located by Games-Howell multiple comparisons test among adhesives for denture materials (p<0.05), between methods for the PMMA only with Olivafix adhesive (p<0.05) and between materials for Olivafix and Fittydent adhesives (p<0.05) (Table 1).

## Discussion

Our study investigated the amount of three different adhesive materials that remain on two different denture base materials after their removal by a brush or a cleansing technique. The study rejected the hypotheses that no differences existed between removal methods, among adhesive materials or between denture base materials.

Regarding the significant differences found among adhesives, results showed that Fittydent was the most difficult adhesive to be removed from both denture base materials, either by the brushing or the cleansing method. For Fittydent, one of the reasons is the high affinity of polyvinylacetate for PMMA,^26,37^ which is the main adhesive component of the material. Perhaps poly(vilyn-methylmethacrylate) component of Olivafix has also a high affinity for PMMA. Another reason for Fittydent is probably the short time the material remained in the bath and for this reason its water-soluble components could not absorb enough amount of water to liquefy the product. This explanation is supported by the results of hydration test (Figure 5), which showed that Fittydent continues to expand 180 min after its wetting, whereas Corega and Olivafix show at this time signs of liquefaction (transparent areas).A higher amount of water-insoluble component in Fittydent than the others or a lower solubility of these may be the explanation of the need of this material for more time in the bath or under running water to loosen its mass from denture surface. Therefore, the material needs to be stored in a water bath or a cleanser longer than the usual time before the brushing action is introduced to the denture surface for its successful removal, especially if this surface is of PMMA.

In respect to the methods, no difference was found between brushing and cleansing action for the removal of Corega and Fittydent adhesives from both denture base materials. However, with the Olivafix, the cleanser was better than the brush to remove the adhesive although from PMMA surfaces. Previous studies have also indicated that the use of a cleanser before or after the use of a brush is effective in removing the adhesive from the denture^27,29^ and that is quite effective in reducing the biofilm that accumulates on denture base surfaces and teeth.^30,38^Although these studies investigated the combined action of a brush with a cleanser, the benefit of the cleanser was evident. In our study, this is also evident; however, it seems that this is not true for all adhesives or cleansers, and depends on adhesive structure and cleanser composition. In our study, Olivafix was the adhesive leaving the least remnants, although not significantly different than Corega, on both denture base surfaces without differences between the methods on the polyamide surface. But on PMMA surface, where the adhesive creates a stronger bond, the cleanser seems to work better than the brush, possible due to the action of sodium lauryl sulphate contained in the cleanser. This is capable of solubilize oil-based components^26,39^, but further investigation on this subject is needed.

Differences between denture base materials were found significant with Olivafix and Fittydent adhesives but not with Corega. The higher presence of adhesive remnants on PMMA surface cannot be explained by the surface roughness of the materials, since Polyamide material was the one with the rougher surface (Figure 3). The higher affinity of the polyvinylacetate^37^or poly(vinyl-methyl-methacrylate) components for PMMA is an explanation. Possibly, the hydrophobic content of the adhesive interacts with non-polar groups of PMMA (such as hydrocarbon chain and methyl groups), enhancing the bonding of the denture adhesive to PMMA surface.^40^We cannot, however, exclude the possibility that the water-insoluble (hydrophobic) content interact with polar groups of PMMA (such as the acetate groups), since it is in fact soluble to the water, although less than 10%,^5^ and differences in this solubility between adhesive products may explain the differences in their removal from PMMA surface. Polyamide is a crystalline polymer in contrast to the PMMA, which is amorphous and has a water sorption and solubility that is lower than that of PMMA due to a low free surface energy and strong hydrogen bond between amide groups.^41^ This is the reason why we cannot expect polar bonds of the hydrophobic component of the denture adhesive and therefore the adhesive is better removed from its surface.

The efficiency of weight measurements of adhesive remnants for estimating the material removal from denture bases is very precise, thus being capable of indicating small differences between different adhesive materials. Brush technique can be standardized; however, it cannot precisely simulate the brushing of this is also one of the limitations of the study, as in most of all laboratory studies. Another limitation would also be the small size of the samples used in place of larger and curved intaglio surface of a denture as to how well these samples represent the actual changes of adhesives in the mouth. Finally, a third limitation of the study is probably the time that the materials remained in the bath. One hour was adequate for the measurements^36^ but still less than 12 h or 24 h, the time that usually an adhesive remains in wearers’ mouth. Therefore, a carefully designed clinical study on patients (with its own limitations) would be more appropriate to answer several removal questions beyond amount of remnants, such as location, location’s morphology, dissolution of materials etc.

Older people have difficulties in keeping their personal hygiene in high levels^42^ and cleaning the denture from adhesive residues is necessary, even with a simple method, since the presence of microorganisms on the denture base inner surface causes inflammation of the oral mucosa. Chemical methods alone, such as the use of peroxide cleanser for denture cleaning are equally or even more effective to brushing alone, and because of its simplicity it is perhaps more useful for those with visual or neuromuscular disorders. However, the method is material and adhesive-depended. Since neither the brushing alone nor the cleanser alone removed completely the adhesive from denture undersurface, to ensure that the quality of life of denture adhesive users improves, finding methods for complete adhesive removal is essential, which also requires further studies.

## Conclusions

Within the limitations of the present *in vitro* study, the following conclusions can be drawn: Fittydent adhesive was the most difficult to be removed, leaving a large amount of material on the surface and needing extra effort and more effective methods for its complete removal. PMMA was also found to be the denture base material with the strongest adherence among the adhesives on its surface requiring additional effort for complete adhesive removal. Finally, denture cleanser found to have a greater effect for removing Olivafix adhesive from PMMA surfaces.
